# Inhibition of Platelet Activation and Thrombus Formation by Adenosine and Inosine: Studies on Their Relative Contribution and Molecular Modeling

**DOI:** 10.1371/journal.pone.0112741

**Published:** 2014-11-13

**Authors:** Eduardo Fuentes, Jaime Pereira, Diego Mezzano, Marcelo Alarcón, Julio Caballero, Iván Palomo

**Affiliations:** 1 Department of Clinical Biochemistry and Immunohematology, Faculty of Health Sciences, Interdisciplinary Excellence Research Program on Healthy Aging (PIEI-ES), Universidad de Talca, Talca, Chile; 2 Centro de Estudios en Alimentos Procesados (CEAP), CONICYT-Regional, Gore Maule, Talca, Chile; 3 Department of Hematology-Oncology, School of Medicine, Pontificia Universidad Católica de Chile, Santiago, Chile; 4 Center for Bioinformatics and Molecular Simulations, Faculty of Engineering in Bioinformatics, Universidad de Talca, Talca, Chile; Irvine, United States of America

## Abstract

**Background:**

The inhibitory effect of adenosine on platelet aggregation is abrogated after the addition of adenosine-deaminase. Inosine is a naturally occurring nucleoside degraded from adenosine.

**Objectives:**

The mechanisms of antiplatelet action of adenosine and inosine *in vitro* and *in vivo*, and their differential biological effects by molecular modeling were investigated.

**Results:**

Adenosine (0.5, 1 and 2 mmol/L) inhibited phosphatidylserine exposure from 52±4% in the control group to 44±4 (p<0.05), 29±2 (p<0.01) and 20±3% (p<0.001). P-selectin expression in the presence of adenosine 0.5, 1 and 2 mmol/L was inhibited from 32±4 to 27±2 (p<0.05), 14±3 (p<0.01) and 9±3% (p<0.001), respectively. At the concentrations tested, only inosine to 4 mmol/L had effect on platelet P-selectin expression (p<0.05). Adenosine and inosine inhibited platelet aggregation and ATP release stimulated by ADP and collagen. Adenosine and inosine reduced collagen-induced platelet adhesion and aggregate formation under flow. At the same concentrations adenosine inhibited platelet aggregation, decreased the levels of sCD40L and increased intraplatelet cAMP. In addition, SQ22536 (an adenylate cyclase inhibitor) and ZM241385 (a potent adenosine receptor A_2A_ antagonist) attenuated the effect of adenosine on platelet aggregation induced by ADP and intraplatelet level of cAMP. Adenosine and inosine significantly inhibited thrombosis formation *in vivo* (62±2% occlusion at 60 min [n = 6, p<0.01] and 72±1.9% occlusion at 60 min, [n = 6, p<0.05], respectively) compared with the control (98±2% occlusion at 60 min, n = 6). A_2A_ is the adenosine receptor present in platelets; it is known that inosine is not an A_2A_ ligand. Docking of adenosine and inosine inside A_2A_ showed that the main difference is the formation by adenosine of an additional hydrogen bond between the NH_2_ of the adenine group and the residues Asn253 in H6 and Glu169 in EL2 of the A_2A_ receptor.

**Conclusion:**

Therefore, adenosine and inosine may represent novel agents lowering the risk of arterial thrombosis.

## Introduction

Cardiovascular diseases (CVD) (i.e., acute myocardial infarction, cerebrovascular disease and peripheral arterial thrombosis) have increased significantly in recent years [1], [2]. The major and independent risk factors for CVD are cigarette smoking, elevated blood pressure, elevated serum total cholesterol and diabetes, among others [3], [4]. Platelet hyper-aggregability is associated with risk factors for CVD [5]. Thus platelets from patients with type 1 and type 2 diabetes exhibit enhanced platelet aggregation activity early [6], [7].

Platelet accumulation at vascular injury sites is the primary event in arterial thrombosis and its activation is a critical component of atherothrombosis [8]. Patients with unstable complex lesions had a fivefold higher expression of platelet activation than patients with stable angina, indicating an intense thrombogenic potential [9]. Also platelets could be directly involved in the unstable plaque through the production and release of pro-inflammatory molecules, including a variety of cytokines, such as TGF-β, IL-1β and sCD40L, among others [10], [11].

Antiplatelet therapy has been used for a long time in an effort to prevent, as well as to treat, thrombotic diseases [12]–[14]. The multiple pathways of platelet activation limit the effect of specific receptor/pathway inhibitors, resulting in limited clinical efficacy [15], [16]. In this way, the best-known inhibitor and turn off signaling in platelet activation is cyclic adenosine monophosphate (cAMP) [17], [18].

Adenosine is a key endogenous molecule that regulates tissue function by activating four G-protein-coupled adenosine receptors: A_1_, A_2A_, A_2B_ and A_3_. Both A_2_ adenosine receptors, A_2A_ and A_2B_, are coupled to G_s_, leading to stimulation of adenylyl cyclase and consequent elevation of cAMP [19]. Adenosine is also recognized as one of the most important endogenous molecules able to prevent tissue injury in ischemia-reperfusion [20]. Inosine is another endogenous purine nucleoside, which is formed during the breakdown of adenosine by adenosine deaminase [21]. Inosine potently inhibited the production of the proinflammatory cytokines (TNF-α, IL-1 and IL-12) and its effect was partially reversed by blockade of adenosine A_1_ and A_2_ receptors [22].

Given the high structural similitude between adenosine and inosine, the main aim of this work was to investigate the relative contribution of these two molecules on platelet activation and thrombus formation. Furthermore, we performed docking experiments on the adenosine receptor A_2A_ in order to explain their differential biological effects at a molecular level.

## Materials and Methods

### Animal research

This study was carried out under recommendations according to the Guide for the Care and Use of Laboratory Animals of the National Institutes of Health. The protocol was approved by the Committee on the Ethics of Animal Experiments of the Universidad de Talca. All efforts were made to minimize suffering.

### Reagents and antibodies

The agonist adenosine 5′- diphosphate bis (ADP), acetylsalicylic acid (ASA), rose bengal, adenosine, inosine and prostaglandin E_1_ (PGE_1_) were from Sigma-Aldrich (St. Louis, Missouri/MO, U.S.A), while the collagen was obtained from Hormon-Chemie (Munich, Germany). Calcein-AM, bovine serum albumin (BSA), SQ22536 (adenylate cyclase inhibitor) and ZM241385 (adenosine receptor A_2A_ antagonist) were obtained from Sigma-Aldrich (St. Louis, Missouri/MO, USA), whereas Luciferase luciferin reagent was obtained from Chrono-Log corp (Havertown, PA) and microfluidic chambers were from Bioflux (Fluxion, San Francisco, California, USA). Annexin V FITC Apoptosis KIT and antibodies (anti-CD62P-PE and anti-CD61-FITC) were obtained from BD Pharmingen (BD Biosciences, San Diego, CA, USA).

### Preparation of human platelet suspensions

After receiving written informed consent from all volunteers, venous blood samples (blood volume of 30 mL was extracted from each volunteer) were taken from six young healthy volunteers (range 20–30 years). The samples were placed in 3.2% citrate tubes (9∶1 v/v) by phlebotomy with vacuum tube system (Becton Dickinson Vacutainer Systems, Franklin Lakes, NJ, USA). The protocol was authorized by the ethics committee of the Universidad de Talca in accordance with the Declaration of Helsinki (approved by the 18th World Medical Assembly in Helsinki, Finland, 1964). Samples obtained from each volunteer were processed independently for each assay and centrifuged at 240 *g* for 10 min to obtain platelet-rich plasma (PRP). Following this, two-thirds of PRP was removed and centrifuged (10 min at 650 *g*). The pellet was then washed with HEPES-Tyrode’s buffer containing PGE_1_ (120 nmol/L). Washed platelets were prepared in HEPES-Tyrode’s buffer at a concentration of 200×10^9^ platelets/L (Bayer Advia 60 Hematology System, Tarrytown, NY, USA). Platelets were kept at 4°C during all the isolation steps after blood samples were taken.

### Flow cytometry analysis for phosphatidylserine externalization and P-selectin exposure

Loss of platelet membrane phopholipid asymmetry with externalization of phosphatidylserine (PS) and P-selectin expression on platelets were determined by flow cytometry [23]. To 480 µL of citrated whole blood, collagen 1.5 µg/mL and ADP 8 µmol/L (final concentrations) were added for 10 min at 37°C, with stirring at 240 *g*. In each experiment, previous to the addition of platelet agonists, the sample was incubated with saline, adenosine (0.5 to 2 mmol/L) or inosine (1 to 4 mmol/L) for 10 min at room temperature. To determine phosphatidylserine externalization, 50 µL of PRP obtained of sample was diluted with 150 µL of binding buffer (10 mmol/L Hepes, 150 mmol/L NaCl, 5.0 mmol/L KCl, 1.0 mmol/L MgCl_2_, 2.0 mmol/L CaCl_2_, pH 7.4) and incubated for 25 min in the dark with 0.6 µg/mL (final concentration) of annexin V-FITC and anti-CD61-PE. To determine platelet P-selectin expression, 50 µL of sample was mixed with saturated concentrations of anti-CD62P-PE and anti-CD61-FITC and incubated for 25 min in the dark. Then, red cells were lysed using FACS lysing solution for 15 min. Samples were then centrifuged and washed for analysis. The samples were acquired and analyzed in Accuri C6 flow cytometer (BD, Biosciences, USA).

Platelet populations were gated on cell size using forward scatter (FSC) vs side scatter (SSC) and CD61 positivity to distinguish them from electronic noise. The light scatter and fluorescence channels were set at logarithmic gain and 5,000 events per sample analyzed. Fluorescence intensities of differentially stained populations were expressed as mean channel value using the cSampler Software (BD Biosciences, USA). All measurements were performed from six separate platelet donors.

### Measurement of platelet secretion and aggregation

Platelet ATP secretion and aggregation were monitored by light transmission according to Born and Cross [24], using a lumi-aggregometer (Chrono-Log, Havertown, PA, USA). Briefly, 480 µL of PRP in the reaction vessel was pre-incubated with 20 µL of saline, PGE_1_ (0.02 mmol/L), adenosine (0.5 to 2 mmol/L) or inosine (1 to 4 mmol/L). After 3 min of incubation, 20 µL of agonist (ADP 8 µmol/L or collagen 1.5 µg/mL) was added to initiate platelet aggregation, which was measured for 6 min. To determine platelet ATP secretion 50 µL of luciferin/luciferase was added within 2 min before stimulation. Platelet ATP secretion and aggregation (maximal amplitude [%]) were determined by software AGGRO/LINK (Chrono-Log, Havertown, PA, USA). Inhibition of the maximal platelet secretion and aggregation was expressed as a percentage with respect to control (saline). The concentration required to inhibit platelet secretion and aggregation by 50% (IC_50_) was calculated from the dose-response curves. All measurements were performed from six separate platelet donors.

### Platelet adhesion and aggregation under controlled flow

Experiments under flow were performed in a BioFlux 200 flow system (Fluxion, San Francisco, California, USA) with high shear plates (48 wells, 0–20 dyne/cm^2^). Using manual mode in the BioFlux software, the microfluidic chambers were coated for 1 hour with 20 µL of collagen 200 µg/mL at a wall shear rate of 200 s^−1^.

The plaque coating was allowed to dry at room temperature for one hour. The channels were perfused with phosphate-buffered saline (PBS) for 10 min at room temperature at wall shear rate of 200 s^−1^ to remove the interface. Then, the channels were blocked with BSA 5% for 10 min at room temperature at wall shear rate of 200 s^−1^. Whole blood anticoagulated with sodium citrate was labeled with calcein-AM (4 µmol/L) and incubated at room temperature with saline, PGE_1_ (0.02 mmol/L), adenosine (0.5 to 2 mmol/L) or inosine (1 to 4 mmol/L). After one hour of incubation, the blood was added to the inlet of the well and chambers were perfused for 10 min at room temperature a wall shear rate of 1000 s^−1^. The plates were mounted on the stage of an inverted fluorescence microscope (TE200, NIKON, Japan) [25].

Platelet deposition was observed and recorded in real-time (30 frames per min) with a CCD camera (QICAM, QIMaging, Surrey, BC, Canada). Bright field and fluorescence microscopy for real-time visualization of platelet adhesion and aggregation in flowing blood was used. For each flow experiment, fluorescence images were analyzed off-stage by quantifying the area covered by platelets with the ImageJ software (version 1.26t, NIH, USA). In each field, the area covered by platelets was quantified. All measurements were performed from six separate platelet donors.

### Effect of ZM241385 and SQ22536 on antiplatelet activity

To investigate whether platelet antiaggregant activity of adenosine and inosine was mediated by stimulation of adenosine receptor A_2A_/adenylyl cyclase pathway, PRP (480 µL at 200×10^9^ platelets/L) was pretreated with SQ22536 (250 µmol/L) or ZM241385 (30 µmol/L) for 3 min. Then, the same PRP was preincubated with 20 µL of adenosine (2 mmol/L) or inosine (4 mmol/L) for 3 min prior to the addition of ADP 8 µmol/L. Platelets were first exposed to ZM241385 or SQ22536 and then ADP was used as controls. All measurements were performed from six separate platelet donors.

### Measurement of cAMP levels in platelets

The effect of adenosine (0.5 to 2 mmol/L) or inosine (1 to 4 mmol/L) on platelet levels of cAMP was evaluated in PRP samples (500 µL) following 5 min incubation without stirring. Reactions were stopped with 150 µL of ice-cold 10% trichloroacetic acid. Precipitated proteins were removed by centrifugation at 2.000 *g* for 15 min at 4°C. Following addition of 150 µL of HCl 1 mol/L, the supernatant was submitted to 6 ether extractions v/v and lyophilized. Samples were stored at −70°C until assay. Before determination, the powder was dissolved in 200 µL of PBS, pH 6.2. cAMP Direct Immunoassay Kit (BioVision Research Products, Mountain View, CA, USA) was employed. All measurements were performed from six separate platelet donors.

### Molecular Docking

Docking was performed using Glide [26], which is contained in Maestro software (Maestro, Version 9.0, Schrödinger, LLC, New York, NY, 2007). Glide docking uses a series of hierarchical filters to find the best possible ligand binding locations in a previously built receptor grid space. The filters include a systematic search approach, which samples the positional, conformational, and orientational space of the ligand before evaluating the energy interactions between the ligand and the protein.

The coordinates of the adenosine receptor A_2A_ were extracted from the X-ray crystal structure of human A_2A_R with adenosine bound (accession code in Protein Data Bank (PDB): 2YDO). The structures of adenosine and inosine were sketched with Maestro software. The extra-precision (XP) module of Glide was used. A grid box of 30Å × 30Å × 30Å was first centered on the center of mass of the adenosine in PDB 2YDO. Default docking parameters were used [27]. The docking hierarchy began with the systematic conformational expansion of the ligand followed by placement in the receptor site. Then minimization of the ligand in the field of the receptor was carried out using the OPLS-AA [28] force field with a distance-dependent dielectric of 2.0. Afterward, the lowest energy poses were subjected to a Monte Carlo procedure that samples the nearby torsional minima. The best pose for a given ligand was determined by the Emodel score, while different compounds were ranked using GlideScore [29]. The docking poses for both ligands were analyzed by examining their relative total energy score. The more energetically favorable conformations were selected as the best poses.

### Thrombus formation in murine model

All studies were approved by the committee on animal care and conform to the Guide for the care and use of Laboratory Animals of Universidad de Talca. The Thrombosis in mice was performed by photochemical injury using a modification of the model described by Przyklenk and Whittaker [30]. For murine model we used the same mice species (C57BL/6) and gender (male) for both in control and experimental groups. Briefly, mice (12–16 weeks old) were anesthetized with a combination of tribromoethanol (270 mg/kg) and xylazine (13 mg/kg), before anesthetizing the animals; the mice were carefully handled to minimize stress and quickly anesthetized. Twenty-four hours prior to surgery, systolic blood pressure was measured using a noninvasive blood pressure meter (BP-98A, Softron Co. Ltd., Tokyo, Japan) in awake mice and during thrombosis. Throughout the procedure, animals were kept on a heating pad maintained at 37°C. After the administration of anesthesia, the mice were placed in the supine position and the mesentery was exposed by central incision in the abdomen, permitting visualization of thrombus development in mesenteric artery. The mice were injected with rose bengal through tail vein injection in a volume of 0.1 mL at a concentration of 50 mg/kg. Just after injection, a 1.5-mW green light laser (532 nm) was applied to the desired site of mesenteric artery and blood flow was monitored for 60 min. Stable occlusion was defined as a blood flow of 0 mL/min for 3 min. Saline (control group, n = 6), ASA (200 mg/kg, n = 6), adenosine (200 mg/kg, n = 6) or inosine (200 mg/kg, n = 6) was administered intraperitoneally 30 min before experiment. After laser exposure, the image of the injury generated of the injured vessel was recorded with a charge-coupled device camera (Optronics, Goleta, CA). The recorded video image was digitized and then analyzed with ImageJ software (version 1.26t, NIH, USA) [31]. In control experiments, neither injection of rose bengal without green laser or without rose bengal with green laser, resulted in any alterations in blood flow (data not shown).

### Measurement of sCD40L levels

Soluble CD40 ligand (sCD40L) was determined using a Human sCD40-Ligand Quantikine kit (R & D systems, Minneapolis, MN). Briefly, washed platelets (200×10^9^ platelets/L) were pretreated with saline, ASA (0.3 mmol/L), adenosine (0.5 to 2 mmol/L) or inosine (1 to 4 mmol/L) for 15 min at 37°C and then stimulated by thrombin (2 U/mL) for 45 min at 37°C. Finally, the supernatants were collected following centrifugation at 11.000 *g* for 10 min at 4°C and stored at −70°C prior to sCD40L measurements by ELISA as described earlier [32]. All measurements were performed from six separate platelet donors.

### Statistical analysis

Data were analyzed using SPSS version 17.0 (SPSS, Inc., Chicago, Illinois. and expressed as mean ± standard error of mean (SEM). Six or more independent experiments were performed for the different assays. Results were expressed as percent inhibition or as percentage of control (as 100%). Fifty-percent inhibitory concentration (IC_50_) of adenosine or inosine against agonist-induced platelet function was calculated from the dose-response curves. Differences between groups were analyzed by Student’s t-test or one-way analysis of variance (ANOVA) using Tukey's post-hoc test. P values <0.05 were considered significant.

## Results

### *In vitro* findings antiplatelet effects

#### Effects of adenosine and inosine on platelet activation events

**Figure 1:**
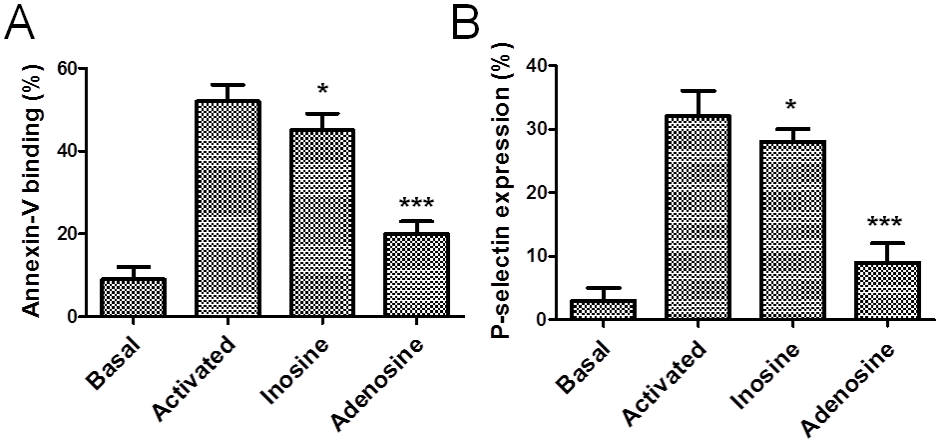
Effects of adenosine and inosine on platelet activation. Blood sample was incubated with saline, adenosine (2 mmol/L) or inosine (4 mmol/L) for 10 min, prior to measuring human platelet activation induced by agonists (collagen/ADP). Phosphatidylserine externalization (n = 9 experiments, panel A) and P-selectin expression (n = 6 experiments, panel B) were determined by flow cytometry. The basal bar represents the fluorescence in unstimulated sample. The graph depicts the mean ± SEM, *p<0.05 and ***p<0.001. The results presented are from 6 separate volunteers (each donors performed as single triplicates).

The antiplatelet effects of adenosine and inosine were explored by testing their activity on different activation-dependent events in human platelets. Activated platelets expose PS, which is a key phenomenon for generating a burst of thrombin essential for thrombus growth. Collagen/ADP-induced externalization of PS assessed by annexin-V binding in the presence of adenosine 0.5, 1 and 2 mmol/L was inhibited from 52±4% in the control group to 44±4% (p<0.05), 29±2 (p<0.01), and 20±3% (p<0.001), respectively. Whereas collagen/ADP-induced externalization of PS assessed by annexin-V binding was only slightly inhibited by 4 mmol/L of inosine (p<0.05) (Figure 1A). The influence of adenosine (0.5 to 2 mmol/L) and inosine (1 to 4 mmol/L) on P-selectin expression by human platelet after stimulation of ADP/collagen in citrated whole blood was measured by flow cytometry. P-selectin expression in the presence of adenosine 0.5, 1 and 2 mmol/L was inhibited from 32±4 to 27±2 (p<0.05), 14±3 (p<0.01) and 9±3% (p<0.001), respectively. At the concentrations tested, inosine only to 4 mmol/L has effect on platelet P-selectin expression (p<0.05) (Figure 1B).

#### Effects of adenosine and inosine on platelet ATP secretion and aggregation

**Figure 2:**
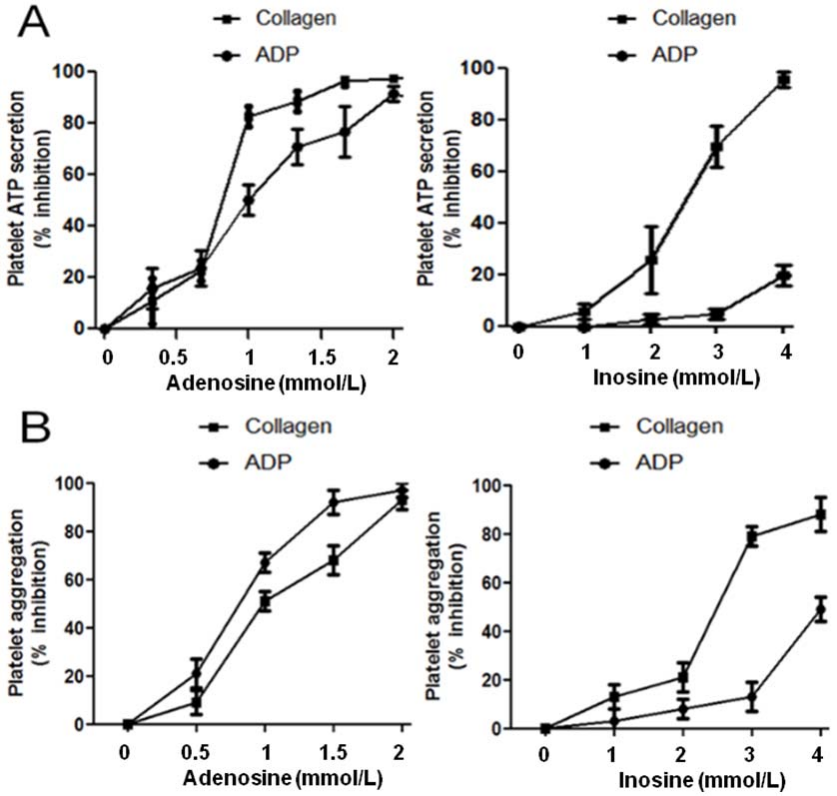
Effects of adenosine and inosine on ADP (8 µmol/L), collagen (1.5 µg/mL), induced platelet ATP secretion (A) and aggregation (B). PRP was pre-incubated with saline (control), adenosine (0.5 to 2 mmol/L) or inosine (1 to 4 mmol/L). After 3 min of incubation, 20 µL of agonist (ADP 8 µmol/L or collagen 1.5 µg/mL) was added to initiate platelet aggregation, which was measured for 6 min. The inhibition of the maximal platelet ATP secretion and aggregation were expressed as a percentage with respect to control (mean ± SEM; n = 6). The results presented are from 6 separate volunteers (each donors performed as single triplicates).

The effects of adenosine and inosine on platelet ATP secretion induced by ADP and collagen are shown in Figure 2A. Adenosine inhibited ADP-induced ATP secretion with a 50% inhibitory concentration (IC_50_) of 0.96 mmol/L. Similarly, the IC_50_ concentration for adenosine on collagen-induced platelet ATP-secretion was about 0.78 mmol/L. Moreover, inosine effectively inhibited the collagen-induced platelet ATP secretion with an IC_50_ of 2.3 mmol/L.

The effects of adenosine and inosine on ADP- and collagen-induced platelet aggregation are shown in Figure 2B. Adenosine effectively reduced ADP-induced platelet aggregation with an IC_50_ of 0.53 mmol/L. Similarly; adenosine suppressed collagen-induced platelet aggregation with an IC_50_ of 0.87 mmol/L. Moreover, inosine effectively inhibited collagen-induced platelet aggregation with an IC_50_ of 2.38 mmol/L. In addition, inosine showed only a mild inhibitory effect (46±5%, p<0.05) over ADP-induced platelet aggregation at a concentration of 4 mmol/L.

#### Adenosine and inosine impair platelet adhesion on immobilized collagen under flow conditions

**Figure 3:**
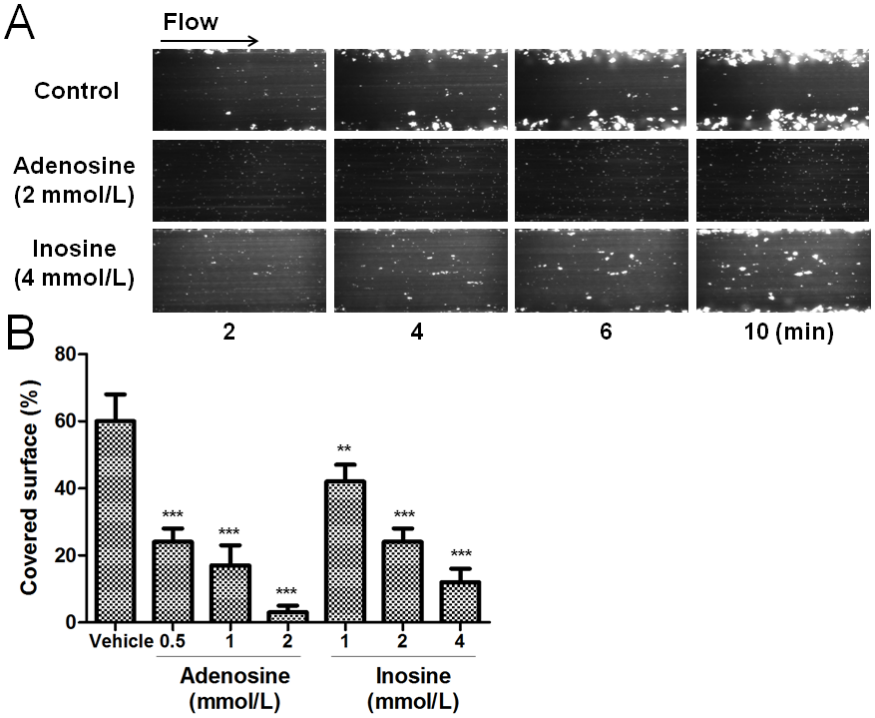
Effects of adenosine and inosine on collagen-induced platelet adhesion and aggregation under arterial flow conditions. Panel A shows the time-lapse of 10 min at 10 dyne/cm^2^. Panel B shows the surface covered by platelets expressed as the percentage of the total surface observed, values are mean ± SEM; n = 6. **p<0.01 and ***p<0.001. The results presented are from 6 separate volunteers (each donors performed as single triplicates).

The effects of adenosine and inosine on platelet adhesion/aggregation to immobilized collagen under arterial flow conditions are shown in Figure 3. After perfusion of citrate-anticoagulated blood over collagen coated plaque surfaces at 37°C with a wall shear rate of 1000 s^−1^ for 10 min, rapid platelet adhesion and aggregate formation were observed (Figure 3A). Adenosine (0.5 to 2 mmol/L) and inosine (1 to 4 mmol/L) concentration-dependently reduced collagen-induced platelet adhesion and aggregate formation under controlled flow. As shown in Figure 3B, platelet adhesion and aggregate formation under controlled flow in presence of adenosine 0.5, 1 and 2 mmol/L was inhibited from 60±8 to 24±4 (p<0.001), 17±6 (p<0.001) and 3±2% (p<0.001), respectively. Similarly, in presence of inosine 1, 2 and 4 mmol/L platelet adhesion and aggregate formation under controlled flow was inhibited from 60±8 to 42±5 (p<0.01), 24±4 (p<0.001) and 12±4% (p<0.001), respectively.

### Mechanisms involved

#### Effect of ZM241385 and SQ22536 on ADP-induced platelet aggregation

**Figure 4:**
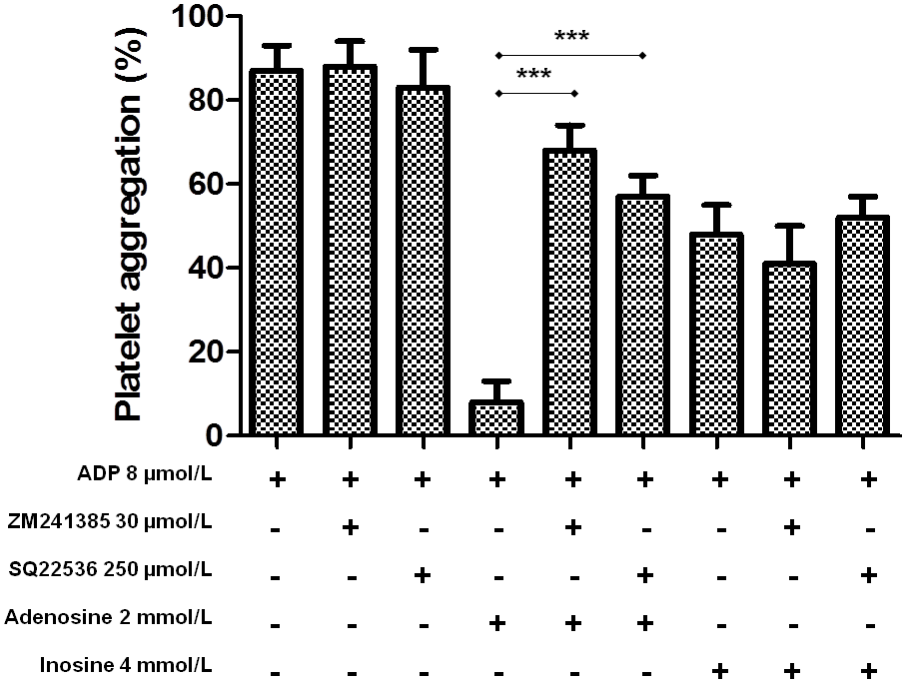
Effect of adenosine receptor A_2A_ antagonist (ZM241385) and adenylyl cyclase inhibitor (SQ22536) on ADP-induced platelet aggregation. PRP suspension was incubated with ADP, adenosine or inosine plus ADP or pretreated with SQ22536 or ZM241385 for 3 min, followed by addition of adenosine or inosine and ADP. The graph depicts the mean ± SEM of n = 6 experiments. ***p<0.001. The results presented are from 6 separate volunteers (each donors performed as single triplicates).

We tested whether ZM241385 and SQ22536 could reverse the inhibitory effect of adenosine and inosine on platelet aggregation induced by ADP. As shown in Figure 4, ZM241385 (30 µmol/L) and SQ22536 (250 µmol/L) attenuated the inhibitory effect of adenosine against ADP-induced platelet aggregation from 8±5 to 68±6 and 57±5%, respectively (p<0.001). In addition, we found that SQ22536 attenuated an increase of intraplatelet levels of cAMP by adenosine from 29±2 to 9±1 pmol/10^8^ platelets (p<0.05). On the contrary, ZM241385 and SQ22536 did not have any effect on the platelet antiaggregant activity of inosine (1 to 4 mmol/L). ZM241385 and SQ22536 alone did not exert any effect on ADP-induced platelet aggregation and with no statistical differences between them.

#### Effects of adenosine and inosine on intraplatelet levels of cAMP

**Figure 5:**
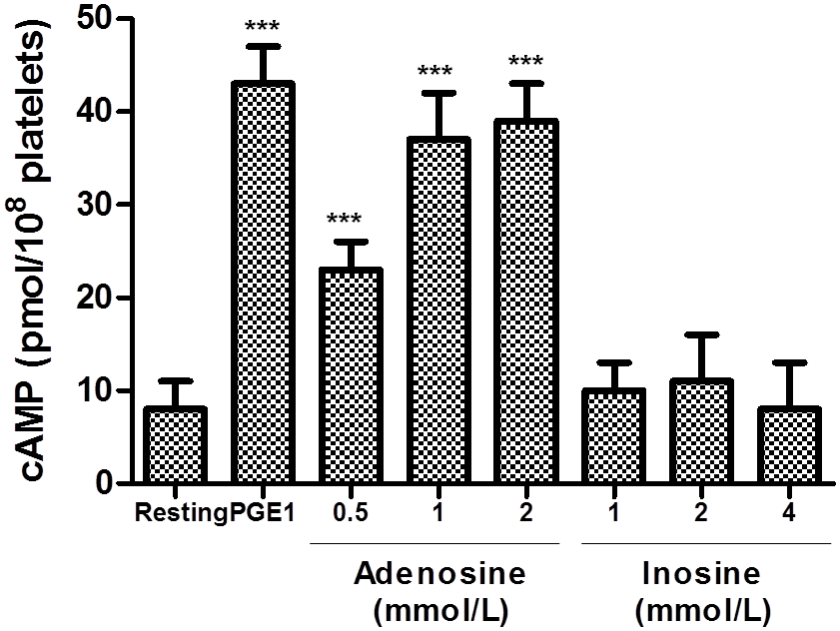
Effects of adenosine and inosine on intraplatelet levels of cAMP. Platelets were incubated with PGE_1_ (0.02 mmol/L, positive control), adenosine (0.5 to 2 mmol/L) or inosine (1 to 4 mmol/L) for measurement of cAMP formations as described in *Materials and methods*. ***p<0.001 as compared with resting platelets (n = 6). The results presented are from 6 separate volunteers (each donors performed as single triplicates).

We investigated whether the effects of adenosine and inosine on platelet function were mediated by changes in platelet cAMP levels. As shown in Figure 5, levels of cAMP in resting platelets were significantly lower compared with PGE_1_ (0.02 mmol/L)-treated platelets (p<0.001). At the same concentrations adenosine significantly inhibits platelet aggregation and concentration-dependently (0.5 to 2 mmol/L) increased the intraplatelet levels of cAMP (p<0.001). On the other hand, inosine (1 to 4 mmol/L) did not show any effect on intraplatelet levels of cAMP.

#### Molecular docking

**Figure 6:**
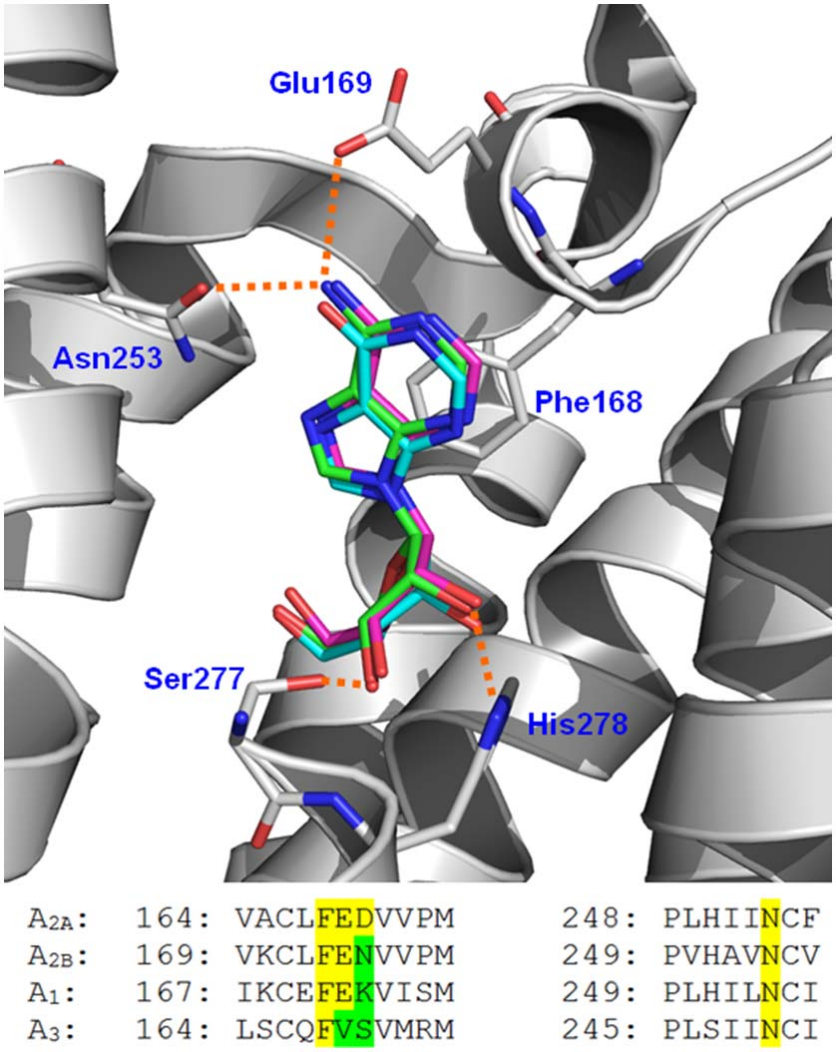
Molecular modeling of adenosine and inosine on adenosine receptor A2A. (top) Molecular conformations of adenosine (green) and inosine (cyan) obtained using docking inside adenosine receptor A2A binding pocket are represented. The X-ray reference structure of adenosine is represented in purple (PDB code: 2YDO). A comparison between the conformations reflects that inosine and adenosine adopt the same binding orientation inside adenosine receptor A2A (bottom). Alignment of the A_2A_ motifs that contain residues Glu169 and Asn253 with A_2B_, A_1_, and A_3_.

Figure 6 shows the alignment of the cocrystallized adenosine structure versus the docked structures for adenosine and inosine. According to this figure, the docked adenosine structure fitted in an optimal way with the adenosine in the crystal structure, and the inosine had the same orientation inside the adenosine receptor A_2A_ binding pocket. Both compounds contain a ribose group that extends deep into the ligand-binding pocket where it has hydrogen bond (HB) interactions with the residues Ser277 and His278 in H7. In addition, both compounds have a π-stacking interaction with Phe168 in extracellular loop 2 (EL2).

Adenosine forms an additional HB between the NH_2_ of the adenine group and the residues Asn253 in H6 and Glu169 in EL2 of the adenosine receptor A_2A_, whereas the hypoxanthine group of inosine cannot establish these interactions. The role of Glu169 in ligand binding has been identified previously in the literature [33], suggesting that it is either directly or indirectly involved in the molecular recognition of both adenosine agonists and antagonists [33].

### *In vivo* assessment of the antithrombotic potential

#### Effect on arterial thrombus formation *in vivo*

**Figure 7:**
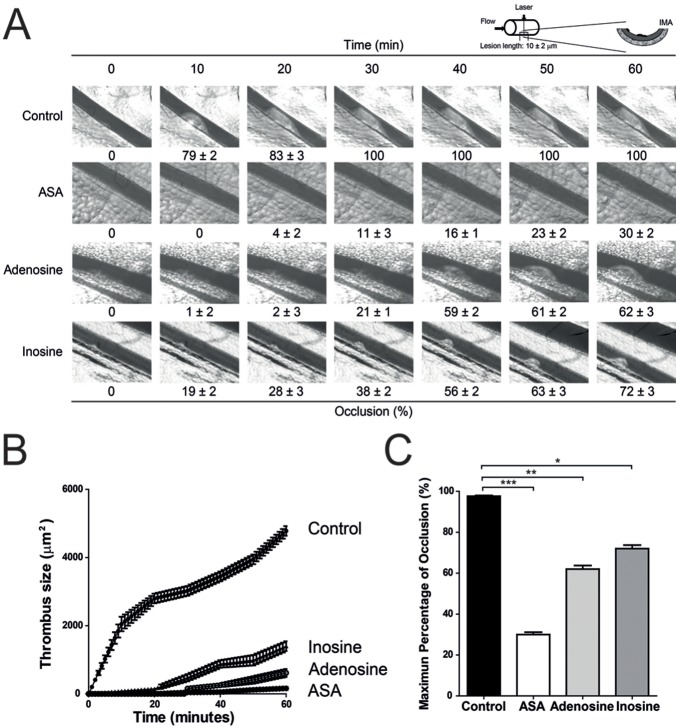
Adenosine and inosine inhibited arterial thrombosis formation. Panel A shows thrombus formation after laser irradiation in the saline control group (n = 6); ASA (acetylsalicylic acid) (200 mg/kg, n = 6); adenosine (200 mg/kg, n = 6) and inosine (200 mg/kg, n = 6). The figures below each photograph represent the percentages of occlusions caused by thrombus formation. Panel B shows the time course changes of thrombus size (µm^2^). Panel C shows maximum percentage of occlusion at 60 min after laser irradiation. For panels A and C the percentages of occlusions were calculated using the percentage of a certain area where the occlusion is greater. The graph depicts the mean± SEM of n = 6 experiments. *p<0.05, **p<0.01 and ***p<0.001.

To study arterial thrombus development in mesenteric artery, anesthetized animals received saline, ASA, adenosine or inosine at a dose of 200 mg/kg body weight by intraperitoneal injection. The effect of adenosine and inosine on arterial thrombus formation was examined and is shown in Figure 7A. Both adenosine and inosine showed a different kinetic inhibition on arterial thrombus formation (Figure 7B). The thrombotic vessel occlusion at 60 min was inhibited from 98±2 to 30±1.8% (n = 6, p<0.001) by pretreatment with ASA. Similarly, adenosine and inosine significantly inhibited arterial occlusion. Administration of adenosine showed a significant reduction in occlusion extent compared with the negative control at 60 min (62±2 vs 98±2%, respectively, n = 6) (p<0.01). On the other hand, inosine exhibited a minor effect on occlusion size reduction from 98±2 to 72±1.9% (n = 6, p<0.05) (Figure 7C).

### Additional anti-inflammatory properties

#### Effect on the levels of sCD40L

**Figure 8:**
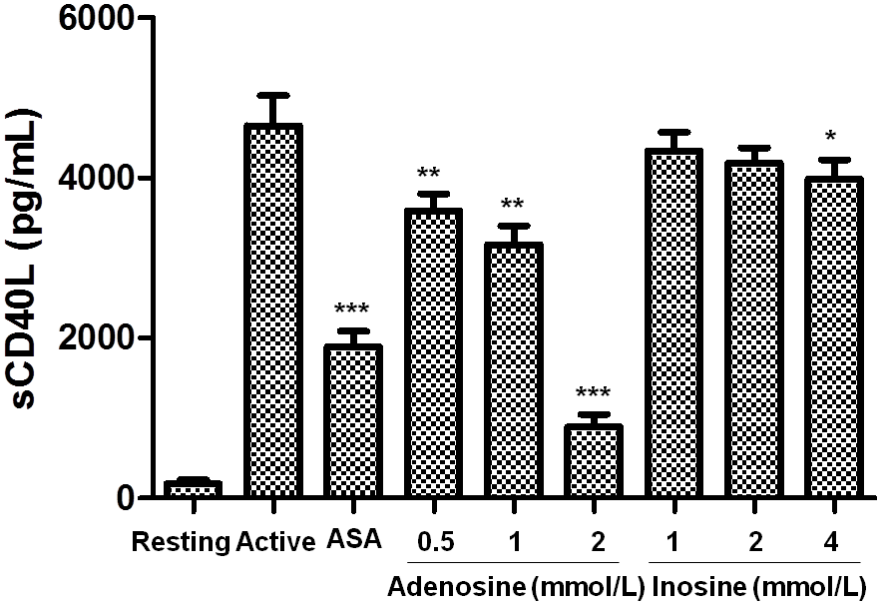
Effect of adenosine and inosine on release of sCD40L from platelets. Washed platelets were incubated with saline, adenosine, inosine or ASA (acetylsalicylic acid) and then stimulated with thrombin. The graph depicts the mean± SEM of n = 6 experiments. *p<0.05, **p<0.01 and ***p<0.001 as compared with the active (saline and then stimulated with thrombin). The results presented are from 6 separate volunteers (each donors performed as single triplicates).

As platelets are considered the major source of sCD40L in blood, we examined the effect of adenosine and inosine on the release of sCD40L. As observed in Figure 8, we found that adenosine (0.5 to 2 mmol/L) concentration-dependently reduced thrombin-induced sCD40L released from platelets (p<0.001). Adenosine exhibited a similar effect as ASA on sCD40L release. Moreover, inosine has a residual effect only at 4 mmol/L over sCD40L released from platelet (p<0.05).

## Discussion

In this study, we have demonstrated that adenosine and inosine display *in vitro* and *in vivo* antiplatelet activities and reduce platelet release of atherosclerotic-related inflammatory mediator (sCD40L). This inhibitory effect of adenosine and inosine was demonstrated with the use of different agonists (ADP and collagen) and this inhibition was directly proportional to the concentrations used.

Adenosine is a natural product and endogenous nucleoside with antiplatelet activity [34]–[36]. Adenosine through G-protein linked receptors activate adenylate cyclase and increase cellular cAMP levels, showing inhibition of platelet function [37], [38]. Studies have established that the inhibitory effect of adenosine on platelet aggregation disappears after the addition of adenosine-deaminase [39], [40], and converts the purine nucleoside into inosine [41]. However, our results show that inosine possesses antiplatelet activity *in vitro* by significantly inhibiting platelet function (activation, secretion, aggregation and adhesion), releasing sCD40L and for the first time we have demonstrated prevention of thrombus growth *in vivo*. In addition, another study has established that inosine markedly inhibited platelet activation *in vitro* and *in vivo*, as well as cerebral ischemia [42].

Platelet-derived P-selectin seems to contribute to atherosclerotic lesion development and arterial thrombogenesis by forming large stable platelet-leukocyte aggregates [43]. Our results show that adenosine and inosine inhibited P-selectin expression on human platelets induced by ADP/collagen. In this sense, adenosine and inosine could inhibit platelet-leukocyte conjugate formation [44]. It has been shown that the increase of cAMP levels regulates P-selectin expression via activation of PKA [38], [45]. In fact, we have recently demonstrated that an increase in intraplatelet cAMP by adenosine markedly increases the phosphorylation of PKA in human platelets [46].

When platelets adhere to collagen, a ligand-binding–induced signal is generated, leading to platelet spreading that render adherent platelets resistant to shear forces at the site of vascular damage [47]. Interestingly, we found that under conditions of arterial shear, adenosine and inosine significantly reduced platelet adhesion and aggregation to collagen as compared with the negative control.

In the last decade several lines of evidence have supported the notion that the secretion of platelet pro-inflammatory molecules (sCD40L, RANTES, sP-selectin, among others) plays a pathogenic role in both the onset and progression of the atherosclerotic process as well as in thrombotic occlusion of the vessels [48], [49]. Platelets interact with monocytes via a CD40-CD40L-mediated pathway that causes their adherence to the inflamed endothelial layer [50]. Thus, high levels of sCD40L have been associated with platelet activation, suggesting a prognostic marker in patients with advanced atherosclerosis [51]. This study demonstrates for the first time that adenosine and inosine, among its antiplatelet activities, decreases the inflammatory component of activated platelets, by reducing the release of sCD40L.

In this study, ZM241385 (adenosine receptor A_2A_ antagonist) and SQ22536 (adenylyl cyclase inhibitor) were able to attenuate the effect of adenosine on ADP-induced platelet aggregation, but failed to affect the platelet aggregation induced by ADP [52]. These findings suggest that inhibition of platelet aggregation by adenosine is mediated by the stimulation of adenosine receptor A_2A_/adenylate cyclase with increased intraplatelet cAMP concentrations. Adenosine increased cAMP levels and significantly inhibited platelet aggregation stimulated by ADP and collagen. Two recent observations may explain these findings showing that intraplatelet cAMP levels downregulate P2Y1R expression [53] and contribute to maintaining GPVI in a monomeric form on resting platelets [54]. Thus, adenosine through elevation of cAMP suppresses the sCD40L release due to the inhibition of adenylyl cyclase inhibition [46], [55]. However, ZM241385 and SQ22536 were not able to attenuate the effect of inosine on ADP-induced platelet aggregation. Thus inosine by intraplatelet signaling pathways inhibits platelet function (phospholipase C activation rather than PKC) [42]. In this sense, different mechanisms may underlie the antiplatelet activity of adenosine and inosine. In fact, different biological functions of both nucleosides have also been demonstrated [56].

Previously, it has been suggested that adenosine is a natural ligand for the four receptors of the family (A_1_, A_2A_, A_2B_ and A_3_), while inosine is only a weak agonist for the A_1_ and A_3_ adenosine receptors [57]. In addition, A_2A_ and A_2B_ receptors are naturally expressed in platelets [58], [59]. The adenosine receptor A_2A_ has a structure consisting of seven transmembrane helices (H1–H7). The binding modes of adenosine and other adenosine receptor A_2A_ agonists have been reported recently [60]. Despite the high structural similitude and antiplatelet activity between adenosine and inosine, the latter is ineffective at adenosine receptor A_2A_ [60]. We performed docking experiments in order to explain this paradigm at a molecular level. The main difference is that adenosine forms an additional HB between the NH_2_ of the adenine group and the residues Asn253 in H6 and Glu169 in EL2 of the adenosine receptor A_2A_, whereas the hypoxanthine group of inosine cannot create these interactions, therefore losing its activity on the receptor and without an effect on cAMP levels.

In Figure 6 we have also included the alignment of the adenosine receptor A_2A_ sequence motifs that include the A_2A_R residues Glu169 and Asn253 to explain why inosine is not an adenosine receptor A2A ligand, but it is at least a weak agonist for A_1_ and A_3_ adenosine receptors. We can see that Asn253 is conserved in all the adenosine receptors. However, the FED motif in adenosine receptor A2A that contains Glu169 is different in other adenosine receptors. The FED motif is optimal for including adenine, but not optimal for including hypoxanthine. The phenylalanine forms the pi-pi stacking with the purine ring, glutamate forms the HB interaction with NH_2_ of adenine, and aspartate is at the entrance of the pocket (it could be involved in the attraction of the ligand). The phenylalanine is conserved in all the adenosine receptors. However, the glutamate is not conserved in the A_3_ receptor. Since there is no glutamate in the A_3_ receptor, the negative density of the carbonyl oxygens in the hypoxanthine does not perceive a negative charge that prevents its binding. On the other hand, the aspartate at the entrance is mutated by asparagine in the A_2B_ receptor (a neutral amino acid), and by a lysine in the A_1_ receptor (positively charged amino acid). The lysine could contribute to the attraction of the hypoxanthine ring (due to the negative density of hypoxanthine). Summarizing, the changes in FED motif in A_1_ and A_3_ receptors could explain why inosine is at least a weak agonist for these receptors, but there is no evidence that A_1_ and A_3_ receptors are in platelets.

Platelet aggregation plays a key role in the pathogenesis of arterial thrombosis [61]. Therefore, inhibition of platelet aggregation by drugs has been used extensively for the prevention of thromboembolic events especially in patients with acute coronary syndromes. In this study, using a murine model of real-time thrombus formation [62], we demonstrated that adenosine and inosine significantly reduced thrombus growth *in vivo* although to a lesser extent than the effect observed with aspirin.

## Conclusion

Our data supports the notion that the mechanisms underlying antiplatelet activity resulting from the interaction adenosine-adenosine receptor A_2A_ seem to be related to a substantial increase in platelet cAMP levels with inhibition of P-selectin expression, sCD40L release and downregulated downstream signaling pathways of ADP and collagen receptors. On the other hand, although inosine does not seem to interact with platelet receptors it was capable of significantly inhibiting platelet adhesion and aggregation under flow and thrombus growth *in vivo*.
